# A novel mini-open transforaminal lumbar interbody fusion for lumbar degenerative diseases: technical note and preliminary results

**DOI:** 10.1186/s13018-023-04018-7

**Published:** 2023-07-20

**Authors:** Yuhang Ma, Kelv Shen, Xiaozhong Zhou, Peng Zhang, Zhengfeng Lu

**Affiliations:** Department of Orthopedics, the Second Affiliated Hospital of Soochow University, 1055# Sanxiang Road, Suzhou, 215000 Jiangsu China

**Keywords:** Mini-open TLIF, Degenerative lumbar diseases, Paraspinal muscles, Limited subperiosteal dissection

## Abstract

**Background:**

Transforaminal lumbar interbody fusion (TLIF) is an effective and popular surgical procedure for the management of various spinal pathologies, especially degenerative diseases. Surgeons have been pursuing minimally invasive technology as soon as TLIF was appeared. Currently, TLIF can be performed with transforaminal approaches by open surgery, minimally invasive surgery or percutaneous endoscope. We provide a detailed description of a new modified open TLIF with percutaneous pedicle screws, which we refer to as mini-open TLIF. The objective of this study was to present feasibility of this procedure and the preliminary results.

**Methods:**

The study is a prospective study. From January 2021 to March 2022, 96 patients (43 males and 53 females) with neurological symptoms due to degenerative lumbar spine diseases were enrolled. Operation time, blood loss, ambulatory time, hematocrit and complications were recorded during perioperative period. Clinical symptoms were evaluated 1 week, 3 months and 12 months after surgery. Visual analogue scale (VAS) scores for lower back pain and leg pain and Oswestry disability index (ODI) were assessed. Magnetic resonance imaging was performed preoperatively and 12 months postoperatively to emulate cross-sectional area of paraspinal muscles. The lumbar interbody fusion rate was evaluated by CT scanning.

**Results:**

The mean operation time of single level was 112.6 min, and the mean operation time of multilevel was 140.1 min. Intraoperative blood loss of single level was 64.5 ml and was 116.3 ml of multilevel. The VAS and ODI scores before and after surgery were significantly different (*P* < 0.0001) and reached minimal clinically important difference. Atrophy rate of paraspinal muscles was 2.5% for symptomatic side and 1.2% for asymptomatic side. The cross-sectional area before and after the operation and atrophy rate had no statistically significant difference (*P* > 0.05).

**Conclusion:**

Mini-open TLIF is effective and feasible for the treatment of lumbar degenerative diseases especially in multilevel disease, with minor damage to muscle and shorter operation time.

*Trial registration*: This study was performed in line with the principles of the Declaration of Helsinki. Approval was granted by the Ethics Committee of the Second Affiliated Hospital of Soochow University (No. JD-LK2023045-I01).

## Introduction

Transforaminal lumbar interbody fusion (TLIF) has been proven as an excellent treatment option for various spinal pathological conditions, such as degenerative disk disease and deformity [1]. Surgeons have been pursuing minimally invasive technology as soon as TLIF was introduced and many techniques have been developed. Following technical advances in surgical instruments, minimally invasive surgery has attracted considerable interest as an alternative to open surgery. Endoscopic techniques, such as full endoscopic technique and unilateral biportal endoscopic technique, have increased in popularity among surgeons in the past decades. The clinical outcomes among lumbar interbody fusion procedures still remain controversial. Some studies have shown good to early outcomes after endoscopic lumbar interbody fusion. Some surgeons believe that the endoscopic lumbar interbody fusion will become more prevalent and even take the place of open procedure. We consider this to be an incomprehensive opinion. The open surgery can be modified and still has advantages over the next few years. We would like to share a novel mini-open TLIF with a smaller midline incision, limited subperiosteal dissection and percutaneous pedicle screws (PPS), which we refer to as mini-open TLIF (MO-TLIF). The objective of this study was to present the feasibility of this procedure and the preliminary results.

## Materials and methods

### Patient data

This prospective study included 96 patients who suffered from degenerative lumbar disease with single- or multilevel disease (from January 2021 to March 2022). The inclusion criteria were as follows: (1) lumbar degenerative/isthmic spondylolisthesis 1 or 2; (2) lumbar disk herniation with segmental instability; and (3) lumbar foraminal stenosis with segmental instability. Patients were excluded for the following reasons: (1) lumbar spondylolisthesis grade 3 or above; (2) reoperation; (3) bilateral nerve root canal stenosis; and (4) tumors, infections and fractures involving lumbar vertebrae. Patients who were lost to follow-up were excluded.

A total of 47 males and 49 females were recruited in this study, with an average age of 54.8 ± 17.5 years (range 22–89 years). Twenty-one patients were diagnosed with lumbar spondylolisthesis, 45 with lumbar disk herniation with segmental instability and 30 with lumbar foraminal stenosis with segmental instability. Fifty patients had single-segment lesions: L3–L4 in 5 patients, L4–L5 in 24 patients and L5–S1 in 21 patients. Forty-six patients had multi-segment lesions: L3–L5 in 18 patients, L4–S1 in 24 patients and L3–S1 in 4 patients (Table 1). Preoperative symptoms in all patients were unilateral radicular radiation pain with or without intermittent claudication.

**Table 1:** Demographic characteristics of patients

Characterisitics	Values
Mean age (years)	54.8 ± 17.5
Sex (M/F)	47/49
Diagnosis	
Lumbar spondylolisthesis	21
Lumbar disk herniation with segmental instability	45
Lumbar foraminal stenosis with segmental instability	30
BMI	23.9 ± 2.8
Operation level	
Single level	
L3–4	5
L4–5	24
L5–S1	21
Multilevel	
L3–5	18
L4–S1	24
L3–S1	4
Follow-up period (months)	13.2 ± 2.1

### Surgical technique

**Fig. 1:**
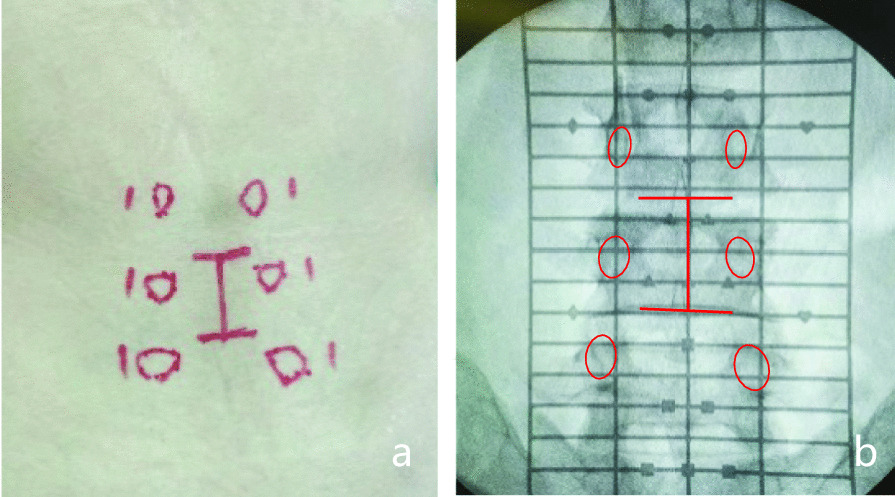
Multilevel mark line on skin. **a** The intervertebral space and projection of pedicle were determined under C-arm fluoroscopy and marked. Then, mark the surgical incision along the spinous process between the two intervertebral spaces and 1.5 cm lateral to the pedicle mark the puncture points of PPS. **b** Corresponding mark position on X-ray

**Fig. 2:**
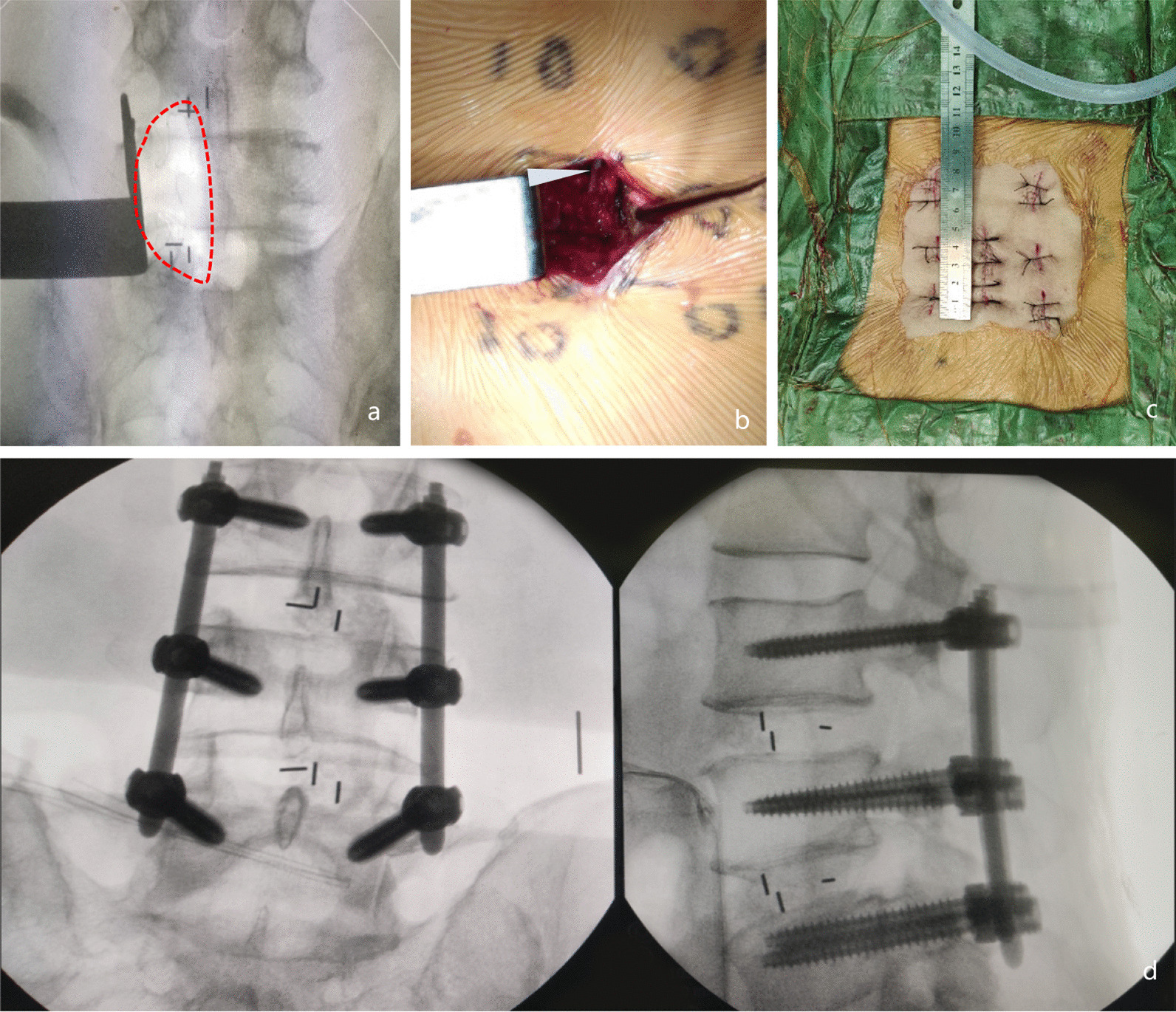
Intraoperative representation of MO-TLIF. **a** Area of dissection for two-level surgery. **b** Intraoperative surgical field and the walking nerve root (triangle). **c** Postoperative photograph showing the incision in MO-TLIF approximately 4 cm for two-segment surgery. **d** Photograph as MO-TLIF completed showing pedicle screws and cages in place

The procedures in all our patients were performed under general anesthesia and took the prone position on a spinal surgery radiolucent table when C-arm fluoroscopy was feasible. Cushions were placed on the chest and hip joints, leaving the abdomen hanging off. This position opened the interlaminar window at the beginning of the procedure by raising the table. Moreover, it helps maintain lumbar lordosis by neutralizing the table at the end of the procedure while installing rods. The entire operation was carried out in two critical steps: (a) decompression, discectomy and cage insertion for interbody fusion and (b) percutaneous placement of pedicle screws. The side of the approach was usually based on the location of the preoperative radicular symptoms. According to the standard anteroposterior position, the incision of the lesion segment and projection of pedicle were determined under C-arm fluoroscopy and marked on the skin (Fig. 1). The surgical site was routinely sterilized and covered with drapes. A posterior midline incision (the incision of single segment is 3 cm and that of two-segment is approximately 4 cm) was introduced for unilateral subperiosteal exposure of the spinous process, lamina and part of facets of the involved segments. One lamina retractor was used to assist with the surgical field exposure. To access the intervertebral space, the entire inferior articular process and part of lamina were identified and resected together, and a part of the superior articular process was resected. Kambin’s triangle can then be clearly observed. Next, the ventral and the excessively thick lateral ligamentum flavum was removed. Discectomy and endplate preparation was carried out. Following this preparation, cage(s) with bone graft was inserted into intervertebral space. Percutaneous pedicle screws and connecting rods were implanted under C-arm fluoroscopy (Fig. 2).

### Clinical assessment

**Fig. 3:**
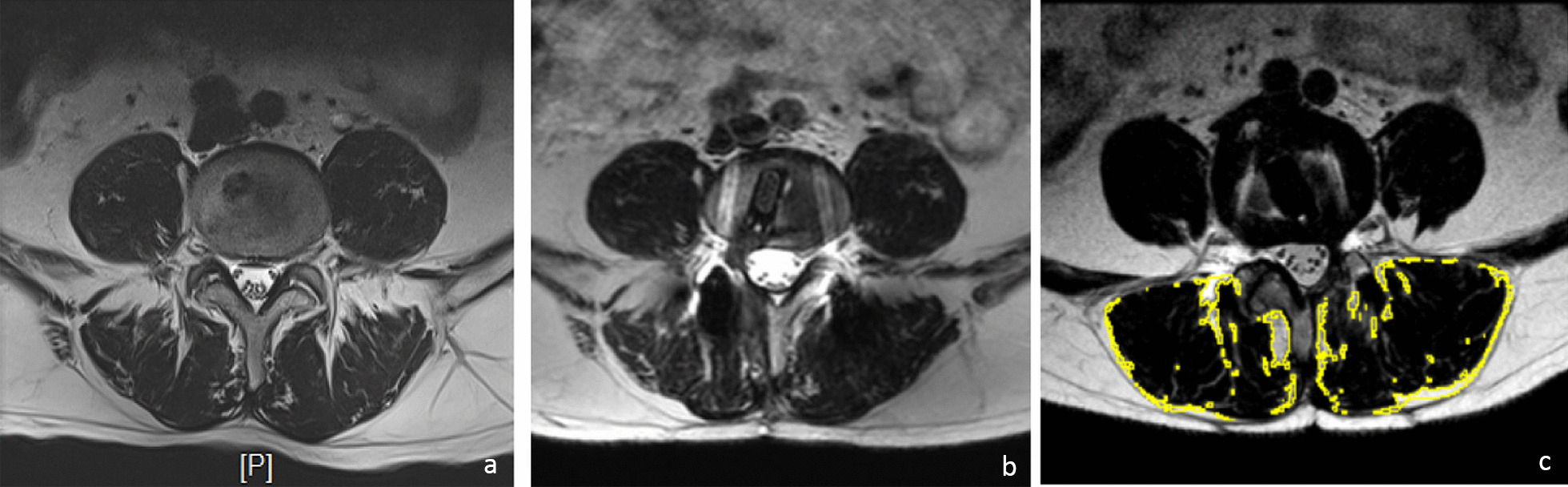
Paraspinal muscles in the same patient before and after operation. **a** Pre-operation. **b** 12 months after operation. **c** Calculating the properties of fat infiltration of paraspinal muscle by ImageJ

The following parameters were recorded: operation time, blood loss, postoperative ambulatory time, follow-up time, complications and visual analogue scale (VAS) and Oswestry disability index (ODI) scores for lumbar and lower extremity pain preoperatively, 1 week postoperatively, 3 months postoperatively and 12 months postoperatively. Preoperative and postoperative hematocrit (Hct), lateral radiography and computed tomography (CT) were used to evaluate intervertebral fusion [2]. Magnetic resonance imaging (MRI) was performed preoperatively and 1 year postoperatively to emulate cross-sectional area (CSA) of segments’ paraspinal muscles. Properties of fat infiltration were calculated by ImageJ (Fig. 3). Cross-sectional area of paraspinal muscles of the lesion segments was emulated and recorded.

### Statistical analysis

VAS and ODI scores before and after surgery were compared by a paired-sample t-test. The cross-sectional area at operational segments before and after operation was compared as well. All statistical analyses were performed using SPSS 27.0(SPSS Inc., Chicago, IL). The data are presented as mean ± standard deviation, and *P* < 0.05 was considered significant.

## Results

This study included 96 patients (47 males and 49 females) with lumbar degenerative diseases, with a mean age of 54.8 ± 17.4 years (range 22–89 years); the demographic characteristics of patients are shown in Table 1. Operation time of single level was 112.6 ± 15.2 min (range 75–160 min), operation time of multilevel was 140.1 ± 16.3 min (range 112–185 min), intraoperative blood loss of single level was 64.5 ± 30.2 ml (range 35–125 ml), intraoperative blood loss of multilevel was 116.3 ± 30.4 ml (range 60–215 ml), and calculated total blood loss in single segment was 366.9 ± 154.8 ml and was 431.2 ± 175.9 ml in multilevel. Postoperative ambulatory time of single segment was 1.7 ± 0.4 days (range 1–3 days), and multilevel ambulatory time was 2.0 ± 0.5 days (range 1–5 days). The perioperative data are shown in Table 2. Follow-up period was 13.2 ± 2.1 months (range 12–18 months). The VAS score for preoperative leg pain was 6.65 ± 0.86, the VAS for preoperative back pain was 5.28 ± 1.19, the VAS for leg pain 1 week postoperatively was 1.67 ± 0.72, the VAS for back pain 1 week postoperatively was 2.34 ± 0.93, the VAS for leg pain 1 year postoperatively was 0.45 ± 0.56, the VAS for back pain 1 year postoperatively was 0.98 ± 0.66, the preoperative ODI (%) was 65.77 ± 10.01, the ODI (%) 1 week postoperatively was 32.95 ± 8.52, and the ODI (%) 1 year postoperatively was 9.38 ± 3.33. The VAS and ODI scores before and after surgery were significantly different (P < 0.0001) and reached minimal clinically important difference (MCID), as shown in Table 3. The CSA for preoperative lumbar muscles on the symptomatic side was 2088.4 ± 226.7 mm^2^ and that on the asymptomatic side was 2081.8 ± 238.6 mm^2^. There was no statistically significant difference. The CSA for lumbar muscles on the symptomatic side postoperatively which was measured by MRI 1 year after operation was 2077.9 ± 225.5 mm^2^ and that on asymptomatic side was 2076.1 ± 235.5 mm^2^. Atrophy rate was 2.5% for symptomatic side and was 1.2% for asymptomatic side, which had no statistically significant difference (*P* > 0.05). The properties of fat infiltration on symptomatic side were 22.14% ± 9.21% preoperatively and 22.09% ± 9.04% postoperatively. The properties of fat infiltration on asymptomatic side were 21.78% ± 8.71% preoperatively and 22.20% ± 9.19% postoperatively. The detailed paraspinal muscle data are presented in Tables 4 and 5. At the last follow-up, 93 (93/96) cases demonstrated solid fusion through CT scanning and flexion–extension lateral radiography, with a fusion rate of 96.88%.

**Table 2:** Perioperative data

	Single level	Multilevel
Pre-op Hct (%)	41.1 ± 4.2	42.4 ± 6.2
Post-op Hct (%)	36.5 ± 4.4	38.1 ± 5.8
Intraoperative blood loss (mL)	64.5 ± 30.2	116.3 ± 30.4
Total blood loss (mL)	366.9 ± 154.8	431.2 ± 175.9
Operation time (min)	112.6 ± 15.2	140.1 ± 16.3
Ambulatory time (days)	1.7 ± 0.4	2.0 ± 0.5

**Table 3:** Preoperative and postoperative outcomes of VAS and ODI scores

Indices	Preoperative	Postoperative			*P* value
		1 week	3 months	1 year	
VAS of back pain	5.28 ± 1.19	2.34 ± 0.93	1.76 ± 0.99	0.98 ± 0.66	< 0.0001
VAS of leg pain	6.65 ± 0.86	1.67 ± 0.72	1.15 ± 0.52	0.45 ± 0.56	< 0.0001
ODI (%)	65.77 ± 10.01	32.95 ± 8.52	18.97 ± 6.51	9.38 ± 3.33	< 0.0001

**Table 4:** CSA and fat infiltration of bilateral paraspinal muscle

	CSA (mm^2^)	Fat infiltration (%)
Preoperative		
Symptomatic side	2088.4 ± 226.7	22.14 ± 9.21
Asymptomatic side	2081.8 ± 238.6	21.78 ± 8.71
Postoperative		
Symptomatic side	2077.9 ± 225.5	22.09 ± 9.04
Asymptomatic side	2076.1 ± 235.5	22.20 ± 9.19

**Table 5:** Paraspinal muscle difference

	Mean ± SD	*P* value
PreSS–PreAS	6.59 ± 36.65	0.081
PreSS–PostSS	10.51 ± 59.68	0.088
PreAS–PostAS	5.77 ± 30.84	0.070
PostSS–PostAS	1.85 ± 80.48	0.822

*PreAS* Preoperative asymptomatic side, *PreSS* preoperative symptomatic side, *PostAS* postoperative asymptomatic side, *PostSS* postoperative symptomatic side

## Discussion

In the past decade, the technologies of tubular retractors and endoscopes have shown dramatic development [3]. Various procedures such as minimally invasive TLIF (MIS-TLIF), percutaneous endoscopic TLIF (PE-TLIF) and unilateral biportal endoscopic TLIF (BE-TLIF) have become popular alternations to conventional open surgery for lumbar degenerative diseases requiring interbody fusion [4–8]. These techniques are superior to traditional open surgery in trauma, bleeding volume and postoperative rehabilitation [9]. Some surgeons believe that the endoscopic lumbar interbody fusion will become more prevalent even take the place of open procedure. However, we proposed that open surgery can be modified and still has advantages. Previous literature demonstrated mini-open TLIF which is similar to MIS-TLIF using Wiltse approach and expandable tubular retractor [10–12]. Our study presents a novel mini-open technique with PPS, smaller midline incision and limited subperiosteal dissection to access the less damage of paraspinal muscles, which is not reported previously as far as we know. MO-TLIF procedure not only aims to acquire similar clinical outcomes and minor damage to muscles compared with other minimally invasive surgeries, such as endoscopic techniques, but also provides a customary alternative for traditional surgeons with a smooth learning curve.

MO-TLIF can be performed with or without visualization aids such as surgical loupes, or microscopes and can handle not only single-level but also multilevel diseases. In the operation of single-level, the paraspinal muscles are subperiosteally dissected from the spinous processes and laminae with a 3 cm incision. In comparison, the systematic review involving 28 studies and 1,475 patients published by Lei Zhu et al. [13] reported that PE-TLIF shows advantages in less surgical trauma and early postoperative relief of back pain and the medium- to long-term clinical outcomes were similar to MIS-TLIF. This meta-analysis shows the mean operative time was 155 min for the PE-TLIF and the mean intraoperative blood loss was 101.1 ml. The mean operative time of MIS-TLIF was 181.1 min, and the mean intraoperative blood loss was 174 ml. The MO-TLIF presents the similar clinical outcomes in terms of VAS scores, ODI scores, bleeding volume, postoperative ambulation time in single-segment treatment and shorter operation time compared with PE-TLIF, which means less surgical trauma than MIS-TLIF. We attempted Wiltse approach in a group of patients previously [14], but it was tough to dissect muscle ideally and took a long time to place tubular retractors, especially in muscular patients, which was not as comfortable as mentioned in certain studies. Meng, Fanjian et al. revealed that there was no significant difference in the deposition of adipose tissue in the paravertebral muscles and the area of paravertebral muscles at the last follow-up (*P* > 0.05) for limited subperiosteal dissection and Wiltse approach [14]. Moreover, MO-TLIF is different from PE-TLIF, which consumes a lot of water for establishing water channel. It would become more meaningful in future if water resources were scarce.

To the best of our knowledge, few studies have reported endoscopic lumbar interbody fusion for multilevel diseases. MO-TLIF can be advantageously applied to treat multilevel lumbar degenerative diseases. The incision can treat the two segments by lengthening 1 cm (to proximally or distally) and 2 cm for three segments. The 49 patients who underwent multilevel operation reveal significant relief of low back pain and leg pain. Preoperatively, most patients presented with intermittent claudication and radicular pain in the lower extremities, and the mean VAS score for leg pain was higher than the mean VAS score for back pain. The postoperative VAS score (back pain and leg pain) and ODI both improved significantly compared to pre-surgery scores. Parker et al. revealed that MCID scores following TLIF are 2.1 points for back pain VAS, 2.8 points for leg pain VAS and 14.9 points for ODI. The VAS and ODI of MO-TLIF reached the MCID scores [15]. The mean operation time of multilevel was 140.1 ± 16.3 min. A slight increase in incision length, blood loss and operation time can achieve the same clinical outcomes as single-segment treatment.

**Fig. 4:**
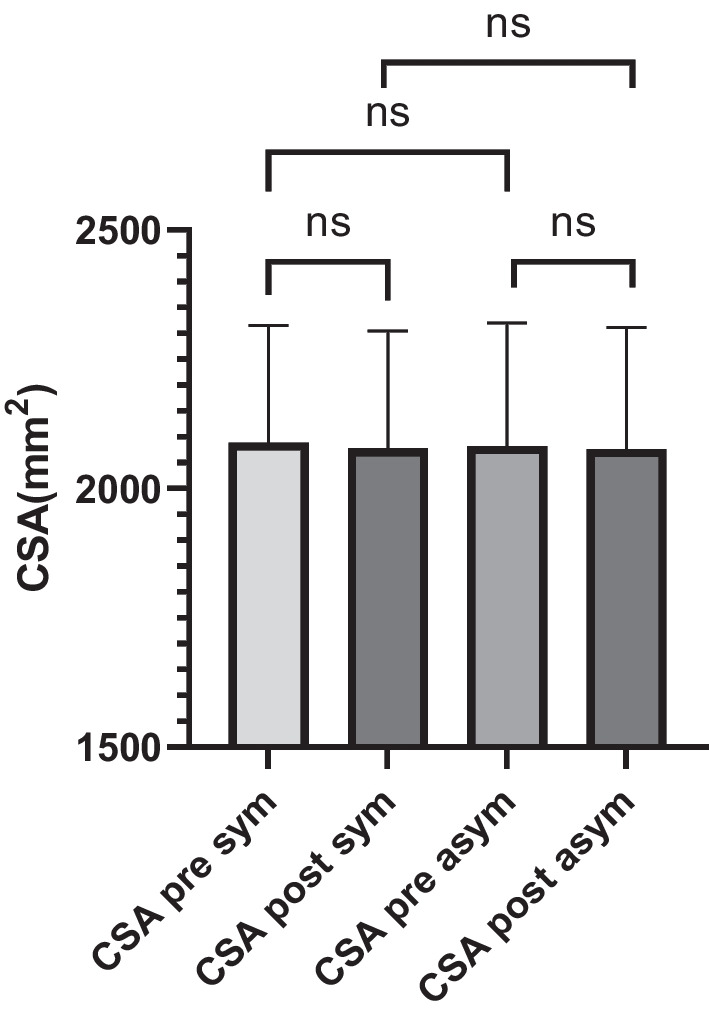
Column chart of preoperative and postoperative CSA

The CSA of symptomatic side (approach side) and asymptomatic side was measured by MRI preoperatively and postoperatively (Table 4). The CSA was measured at the level of the center of the intervertebral disk and calculated the average in multilevel. Preoperative CSA was similar on both sides with no statistical difference (*P* > 0.05; Table 5). The postoperative CSA showed slight atrophy in both sides but was not significant (*P* > 0.05). There are no statistical differences of CSA in postoperatively bilateral paraspinal muscles (Fig. 4). Atrophy rate was 2.5% for symptomatic side and 1.2% for asymptomatic side, which demonstrates that limited subperiosteal dissection did not increase atrophy of paraspinal muscles compared with PPS.

Intraoperative blood loss was estimated by calibrated collection bags, which may have bias. The Nadler’s formula was used to evaluate the blood loss, which takes into account height, weight and sex for calculation [16–22]. However, all blood loss estimation formulas showed a significant tendency to overestimate blood loss according to reference [23]. The total blood loss is 366.9 ± 154.8 mL in single-level operation and 431.2 ± 175.9 mL in multilevel. None of the 96 patients received blood transfusions during the perioperative period.

The main complications included wound infection (*n* = 10), dural tear (*n* = 4) and wrong surgical segment (*n* = 2). No neurological deficit, cage subsidence or screw loosening occurred. It is necessary to reconfirm segment by fluoroscopy after dissection. Ventral and the excessively thick lateral ligamentum flavum were removed. Part of dorsal ligamentum flavum was retained to keep spinal canal intact, thus reducing the possibility of epidural scar formation and shortening the operation time. This technique can also achieve decompression of contralateral central spinal canal and lateral recess by removing the root of spinous process.

This research aims to present a new open TLIF without special surgical instruments and aqueous medium to achieve similar minimally invasive effect compared with PE-TLIF and MIS-TLIF. The most outstanding advantages of MO-TLIF are treatment for multiple segments and amicable learning curve.

This study has some limitations. First, this study did not include a multicenter trial, and there was an absence of a control group. Second, the overall follow-up period was too short (ranging from 12 to 18 months) to determine the clinical efficacy of MO-TLIF. Finally, the blood loss calculation lacks universal assessment methodologies, which may cause a bias.

## Conclusion

Under appropriate patient selection and surgical indications, mini-open TLIF is effective and feasible for the treatment of lumbar degenerative diseases, especially multilevel diseases, with minor muscle damage and shorter operation time. It is an optional method of lumbar interbody fusion.

## Data Availability

The data that support the findings of this study are available from the corresponding author, Zhengfeng Lu, upon reasonable request.

## Abbreviations

TLIF: Transforaminal lumbar interbody fusion
MO-TLIF: Mini-open transforaminal lumbar interbody fusion
MIS-TLIF: Minimally invasive transforaminal lumbar interbody fusion
BE-TLIF: Unilateral biportal endoscopic transforaminal lumbar interbody fusion
PE-TLIF: Percutaneous endoscopic transforaminal lumbar interbody fusion
PPS: Percutaneous pedicle screws
VAS: Visual analogue scale
ODI: Oswestry disability index
CT: Computed tomography
MRI: Magnetic resonance imaging
LBP: Lower back pain
CSA: Cross-sectional area
MCID: Minimal clinically important difference
